# Evaluation of Biological Variation of Different Clinical Laboratory Analytes in the Blood of Healthy Subjects

**DOI:** 10.7759/cureus.36242

**Published:** 2023-03-16

**Authors:** Md. Akshad Ali, Md. Sabir Hossain, Farha Matin Juliana, Md. Selim Reza

**Affiliations:** 1 Pathology Laboratory, United Hospital Limited, Dhaka, BGD; 2 Biochemistry and Molecular Biology, Jahangirnagar University, Dhaka, BGD; 3 Applied Zoology Research Division, Bangladesh Council of Scientific and Industrial Research, Rajshahi, BGD

**Keywords:** reference interval, diagnosis monitoring, reference change value, analytical variation, biological variance

## Abstract

Background

Biological variation (BV) as a prognostic marker implies that each individual has a “subject mean” or central tendency, control level, or “set point” concentration for maintaining homeostasis regulation, which is influenced by factors such as genes, diet, exercise, and age. Uses for information on BV include determining the value of population-based reference intervals, assessing the importance of variation in serial findings, and establishing criteria for judging correct analysis.

Aims

We focused on the assessment of BV parameters for these elements as within-subject BV (CV_W_), between subject BV (CV_G_), the index of individuality (II), and the reference change value (RCV) of important biochemical analytes in the Bangladeshi adult population.

Methodology

This is a cross-sectional analytical study of a representative sample in the population of Bangladesh to determine BV in clinical laboratory analytes. For the study, 758 people were requested to take part; among those 730 (ages 18-65) apparently, healthy adults were blood donors, hospital staff, laboratory personnel, or any individuals who presented themselves for health screening at a tertiary hospital in Dhaka, Bangladesh.

Results

The CV_W_ for blood sugar, creatinine, urea, uric acid, sodium, potassium, chloride, calcium, magnesium, and phosphate were calculated as 5.10 %, 4.64%, 10.72%, 5.71%, 0.69%, 4.35%, 0.75%, 3.69%, 4.57%, and 4.72%, respectively. The CV_G_ for blood sugar, creatinine, urea, uric acid, sodium, potassium, chloride, calcium, magnesium, and phosphate was 10.70%, 21.46%, 31.47%, 23.52%, 1.95%, 9.74%, 2.56%, 4.64%, 9.96 %, and 17.45%, respectively. The index of individuality (II) for blood sugar, creatinine, urea, uric acid, sodium, potassium, chloride, calcium, magnesium, and phosphate were 0.48, 0.22, 0.34, 0.24, 0.35, 0.45, 0.29, 0.79, 0.46, and 0.27, respectively. The RCV for blood sugar, creatinine, urea, uric acid, sodium, potassium, chloride, calcium, magnesium, and phosphate was 14.75%, 14.10%, 30.58%, 16.13%, 2.82%, 12.58%, 3.54%, 10.62%, 13.62 %, and 15.80%, respectively.

Conclusions

Nine serum biochemistry analytes (blood sugar, creatinine, urea, uric acid, sodium, potassium, chloride, magnesium, and phosphate) had low individuality, indicating that subject-based reference intervals are appropriate, only one analyte (calcium) had high individuality and, therefore, population-based reference intervals are more appropriate.

## Introduction

Monitoring the level of glucose in the blood is a crucial component of the method of determining diabetes [1]. Creatinine is a chemical waste product of creatine. Creatinine is synthesized by a nonenzymatic process via the catabolism of creatine, which is required to source energy chiefly for muscles. The kidneys are responsible for filtering the majority of creatinine, while some is actively secreted. Creatinine levels in the blood are an indirect indicator of kidney function because of their association with excretion [2]. Urea is the kidneys' way of getting rid of the nitrogenous waste products of protein metabolism. Decreased urine production due to impaired kidney function increases blood levels of urea [3]. The acid uric acid, which is produced when purine nucleotides are broken down, can be used as a screening tool for many different purine metabolic diseases. Hyperuricemia can lead to cardiovascular disease (CVD), gout, and kidney stones [4]. Electrolytes are vital for cell electrical neutrality, pH regulation, neuron and muscle action potentials, and fundamental life functions. In addition to sodium, potassium, and chloride, other important electrolytes include magnesium, calcium, and phosphate [5]. For those who are especially vulnerable to illness, these clinical measures are essential. For this reason, we determined to use those parameters.

Biological fluids are constantly changing due to the constant movement of their constituents. The term “biological variation” refers to a natural ebb and flow that may be thought of as a random fluctuation around the mean concentration in steady states [6]. Lifespan variation, cyclical variation that might be daily, monthly, or seasonal in character, and random variation are the three categories of biological variation (BV) of human body components investigated in laboratory medicine. Analytical objectives for bias and imprecision are established by distinguishing between intra-individual or within-subject (CV_W_) and inter-individual or between-subject (CV_G_) variability in BV [7]. Moreover, the right application and clinical interpretation of an analyte's result may be gleaned from a thorough understanding of the analyte's BV [8]. Assessments of double samples are performed to evaluate the analytical variation [9]. Imprecision and variations in bias (caused by factors as methodology calibration) are part of analytical variance, although they are typically not noticeable.

All clinical laboratories in Bangladesh use population-general BV to determine analytical targets, the value of reference changes, and the clinical relevance of population-based reference intervals. Hence, health professionals employ BV as a valuable resource for interpreting data and aiding in the decision-making phase, improving patient care in the process. The goals of the study were to determine the biological variance of these clinical parameters and select the most appropriate test for a given clinical scenario.

## Materials and methods

Study design and selection of study subjects

This research study used a representative sample from the population of Bangladesh to determine BV in clinical laboratory analytes. For the study, 758 people were requested to take part; among those 730 (ages 18-65) apparently, healthy adults were blood donors, hospital staff, laboratory personnel, or any individuals who presented themselves for health screening at a tertiary hospital in Dhaka, Bangladesh. So, 730 randomly selected blood donors were recruited for the study, and 730 of them were used to determine between-subject BV (CV_G_) and within-subject BV (CV_W_) by blood sampling on four occasions at 2-week intervals for an appropriate number of people.

 Specimen collection and laboratory analysis

Using a standard vein piercing technique with little stasis, an experienced phlebotomist took all blood samples from sitting individuals. Blood samples were obtained in serum separator tubes for each specimen and sent in ice chests to the lab for examination. After letting the samples coagulate, they were centrifuged at 4,000 g for 10 minutes to remove any contaminants. At the conclusion of the collection period, all serum samples were frozen at -80 degrees Celsius and analyzed as stated or suggested in the literature [7]. After the removal of any extreme data points, statistical analyses will utilize the mean value of an individual's replicated measurements. The same analyst handled all of the tests for each parameter. The pre-analytical variance was deemed to be quite small when using this standardized approach. Blood sugar, urea, creatinine, uric acid, serum sodium, potassium, chloride, calcium, magnesium, and phosphate were among the biochemical analytes evaluated by an automated chemistry analyzer using the photometric and spectrophotometric approaches. When all of the samples were analyzed using the same lots of reagents on the same day, the within-run (intra-assay) analytical variance was reduced to a minimum. To further reduce the potential for inter-assay variance, all samples from a given individual were evaluated simultaneously in the same assay. Analyses of several serum control pools performed in parallel with tests by the research group allowed us to calculate the coefficients of analytical variation (CV_A_).

Data management and analysis

Data that deviate significantly from the rest will be examined using cutoffs of 3 or 4 standard deviations (SD) and 1.5 IQR. By using commercially available statistics software, we determined the mean, standard deviation, and variance of the chosen laboratory data throughout the four visits for each participant. The population's average and standard deviation were determined using these numbers. Combining the BV with the analytical variance yields the overall variation in measurements for four samples from the same subject. The following formula [8] was used to determine the biological differences between subjects or within individuals. (CVw)^2^ = (Total CV)^2^ - (Analytical CV)^2^.

It is claimed that an analytical variance (CV_A_) of less than half the mean within-subject variance (CV_W_)serves as a quality standard for imprecision. The biological and analytic variation that occurs inside an individual is not included in the inter-individual or between-subject variance (CV_G_^2^). Fraser and Harris provided the formula for calculating the biological diversity between people or test subjects [8]. CV_G_ = (CVt^2^ - CV_w_^2^ - CV_A_^2^ )^1/2^.

Most analytes have high degrees of individuality (within-subject BV lower than between-subject BV); therefore, a reference change value (RCV) idea is preferable to the population-based reference interval for identifying shifts in health status [10,11]. Using the collected information, RCV was determined by the following formula, RCV =√ 2 * Z * (C_^A^_^2^ + CVw 2)^1/2 ^[12]. The ratio of the within-subject biological variance (CV_W_) to the between-subject BV (CV_G_) was also used to determine an individuality index (II). We employed IBM SPSS Statistics (Version 26), the R programming language, and Graph Pad Prism version 18 for our statistical research.

Ethics approval and consent to participate

This study was approved by the Ethical Review Committee of Jahangirnagar University, Dhaka, Bangladesh (issued protocol number 6206).

## Results

For the study, 758 people were requested to take part; of those, 730 were enrolled in the research: 436 men (59.726%) and 294 women (40.274%). To set up reference intervals for analytes based on age, the data was roughly split into two age groups (based on less than 40 years and more than 40 years), with 406 (55.616%) of the data coming from participants younger than 40 and 325 (44.52%) coming from participants older than 40 (Table 1).

**Table 1:** Sex and age distribution of participants to evaluation of biological variance

SN	Age group	Total	Male	%	Female	%
1	(18-39)<40	406	251	61.822	155	38.177
2	(40-65)>40	324	185	57.098	139	42.901
3	Total	730	436	59.726	294	40.274

**Figure 1:**
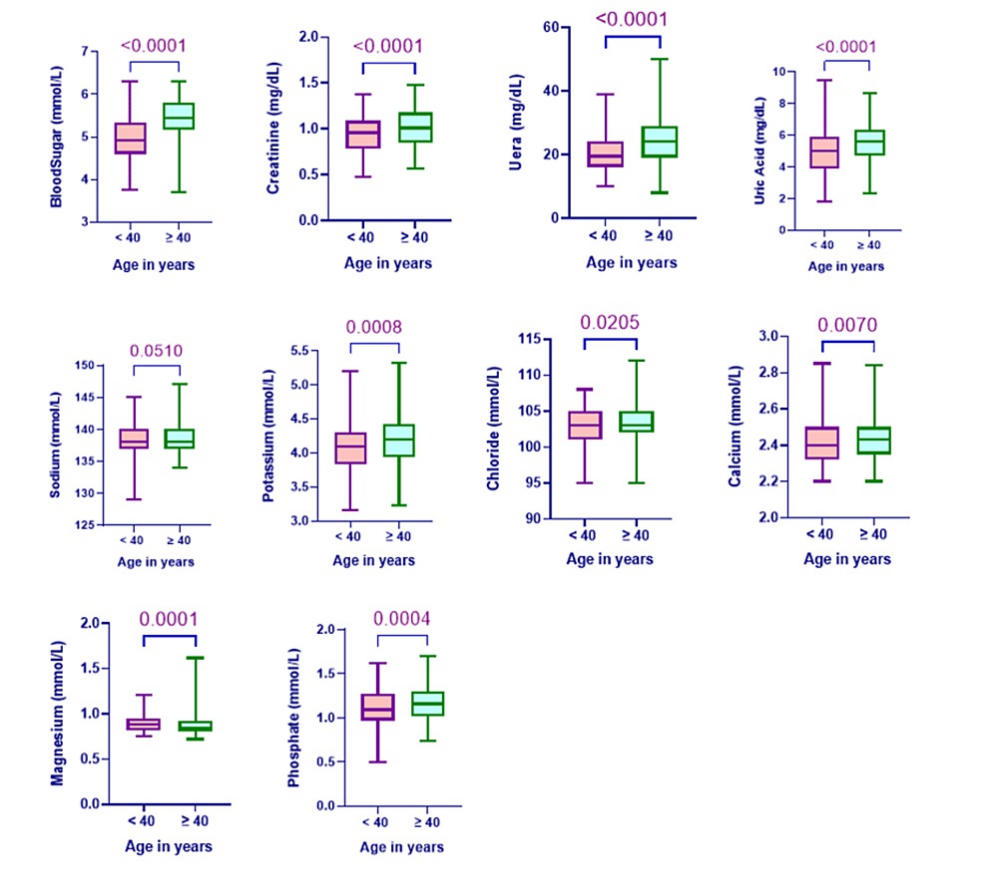
Box and whisker plots showing variation in different clinical parameters with age (<40 Y = less than 40 year, >40 = More than 40 year)

In accordance with the CLSI (NCCLS, 2000) standard for defining reference intervals, the 2.5th and 97.5th percentiles were used to figure out the lower and upper limits of the reference values. U test was used to statistically compare male and female medians. For this study, a p-value of less than 0.05 was deemed significant. Table 2 displayed the median, 95% reference value, 2.5th and 97.5th percentiles, 90% confidence intervals, and p-value of the biochemical analytes for combined men and females, age less than 40, and age more than 40. In Table 2, we found that there are significant sex differences (p > 0.05) in the results of the biochemical tests for glucose, creatinine, urea, uric acid, sodium, chloride, and calcium, but not for potassium, magnesium, or inorganic phosphate. Figure 1 showed statistically significant age differences (p>0.05) in the results of several biochemical tests (blood sugar, creatinine, urea, uric acid, chloride, calcium, potassium, magnesium, and inorganic phosphate), but not in sodium.

**Table 2:** 95% reference values (2.5th and 97.5th percentile values) with their 90% confidence intervals for different clinical parameters CV_G _- between subject biological variations, BS-Blood Sugar, and p<0.05 is consider significant.

Clinical parameters	Subjects	Median	% CV_G_	95% Reference Intervals	90% Confidence Intervals for Lower Limit	90% Confidence Intervals for Upper Limit	P Value
Blood Sugar	Total	5.2	10.70	4.0 to 6.1	3.87- 4.12	6.0 -6.12	
	Males	5.26	10.76	4.01 to 6.1	3.87 - 4.24	6.0- 6.2	0.040
	Females	5.1	10.54	3.99 to 6.1	3.79 - 4.20	6.0- 6.2	
Serum Creatinine	Total	0.98	21.46	0.57 to 1.35	0.53- 0.61	1.33 -1.37	
	Males	1.08	13.76	0.76 to 1.37	0.75 – 0.79	1.35- 1.40	0.000
	Females	0.79	16.67	0.52 to 1.08	0.48 – 0.53	1.04- 1.13	
Serum Urea	Total	21	31.47	11 to 37	10.5-12	35-38.3	
	Males	23	27.07	13 to 38.3	10.86 –13.58	37- 39	0.000
	Females	18	28.88	10.5 to 34	10 – 11	30- 35	
Serum Uric acid	Total	5.23	23.52	2.85 to 7.22	2.7- 2.97	7.14 -7.5	
	Males	5.8	17.89	3.48 to 7.5	2.98–3.87	7.22 - 7.60	0.000
	Females	4.33	23.42	2.5 to 6.41	1.98–2.80	6.2- 6.7	
Serum Sodium	Total	138	1.95	134.8 to 144	134- 136	143 -145	
	Males	139	1.99	134.8 to 145	134 - 136	145- 146	0.001
	Females	138	1.32	134.8 to 142	134 - 136	141- 142	
Serum Potassium	Total	4.12	9.74	3.4 to 5.0	3.36- 3.45	4.9 -5.0	
	Males	4.11	9.93	3.4 to 5.0	3.3 – 3.48	4.9- 5.0	0.532
	Females	4.12	7.80	3.48 to 4.8	3.4 – 3.5	4.7- 5.0	
Serum Chloride	Total	103	2.56	99 to 108	98- 99	107 -108	
	Males	103	2.51	97 to 107	96 – 99	107- 108	0.000
	Females	103.9	2.32	99 to 108	97 – 99	107- 109	
Serum Calcium	Total	2.42	4.64	2.2 to 2.65	2.2- 2.23	2.64 -2.65	
	Males	2.43	4.53	2.2 to 2.65	2.2 – 2.24	2.65-2.65	0.001
	Females	2.40	4.17	2.2 to 2.63	2.2 – 2.23	2.59- 2.65	
Serum Magnesium	Total	0.88	9.96	0.75 to 1.05	0.73- 0.75	1.03 -1.06	
	Males	0.88	9.09	0.75 to 1.06	0.73 – 0.75	1.04- 1.07	0.823
	Females	0.88	7.95	0.75 to 1.04	0.72 – 0.77	1.02- 1.06	
Serum Phosphate	Total	1.11	17.45	0.85 to 1.59	0.81- 0.86	1.46 -1.60	
	Males	1.13	17.39	0.83 to 1.58	0.80- 0.87	1.52 -1.64	0.823
	Females	1.10	15.79	0.87 to 1.59	0.83- 0.88	1.51 -1.62	

The analytical variation (CV_A_) for blood sugar, creatinine, urea, uric acid, sodium, potassium, chloride, calcium, magnesium, and phosphate were 1.53%, 2.09%, 2.61%, 1.13%, 0.75%, 1.29%, 1.04%, 1.04%, 1.81%, and 3.20%, respectively. The intra biological variance (CV_W_) for blood sugar, creatinine, urea, uric acid, sodium, potassium, chloride, calcium, magnesium, and phosphate were calculated as 5.10%, 4.64%, 10.72%, 5.71%, 0.69%, 4.35%, 0.75%, 3.69%, 4.57%, and 4.72%, respectively. The intra biological variance CV_G_s for blood sugar, creatinine, urea, uric acid, sodium, potassium, chloride, calcium, magnesium, and phosphate were 10.70%, 21.46%, 31.47%, 23.52%, 1.95%, 9.74%, 2.56%, 4.64%, 9.96%, and 17.45%, respectively. The index of individuality (II) and RCV of the clinical parameters were calculated in Table 3. For blood sugar, creatinine, urea, uric acid, sodium, potassium, chloride, calcium, magnesium, and phosphate, the three levels of models for quality specifications such as minimum, desirable, and optimal analytical goals for imprecision, bias, and total error derived from the biological data are shown in Table 4.

**Table 3:** Estimated variance of components for clinical parameters derived from data on biological variation CV_A_- coefficient of variation of analytical levels; CV_W_ - coefficient of variation of within-subject levels; CV_G_ - coefficient of variation of between-subject levels; II - an index of individuality; RCV - reference change value

Clinical Parameters	CV_A_ %	CV_W _%	CV_G_ %	II	RCV %
Blood Sugar	1.53	5.10	10.70	0.48	14.75
Serum Creatinine	2.09	4.64	21.46	0.22	14.10
Serum Uera	2.61	10.72	31.47	0.34	30.58
Serum Uric Acid	1.13	5.71	23.52	0.24	16.13
Serum Sodium	0.75	0.69	1.95	0.35	2.82
Serum Potassium	1.29	4.35	9.74	0.45	12.58
Serum Chloride	1.04	0.75	2.56	0.29	3.54
Serum Calcium	1.04	3.69	4.64	0.79	10.62
Serum Magnesium	1.81	4.57	9.96	0.46	13.62
Serum Phosphate	3.20	4.72	17.45	0.27	15.80

**Table 4:** Quality specifications for clinical biomarkers measurement derived from our data on biological variation TEa - Allowable Total Error

Clinical Parameters	Quality level	Imprecision, %	Bias, %	TEa%
Blood Sugar	Optimal	1.274	1.48	3.58
	Desirable	2.5	2.96	7.17
	Minimal	3.822	4.45	10.75
Serum Creatinine	Optimal	1.16	2.74	4.65
	Desirable	2.32	5.48	9.31
	Minimal	3.48	8.23	13.97
Serum Urea	Optimal	2.68	4.15	8.58
	Desirable	5.36	8.31	17.15
	Minimal	8.04	12.46	25.73
Serum Uric acid	Optimal	1.43	3.02	5.38
	Desirable	2.85	6.04	10.76
	Minimal	4.28	9.07	16.14
Serum Sodium	Optimal	0.17	0.25	0.54
	Desirable	0.34	0.51	1.08
	Minimal	0.51	0.77	1.62
Serum Potassium	Optimal	1.08	1.33	3.12
	Desirable	2.17	2.67	6.25
	Minimal	3.26	4.00	9.38
Serum Chloride	Optimal	0.19	0.33	0.64
	Desirable	0.37	0.67	1.28
	Minimal	0.56	1.00	1.92
Serum Calcium	Optimal	0.92	0.74	2.26
	Desirable	1.84	1.48	4.52
	Minimal	2.76	2.22	6.78
Serum Magnesium	Optimal	1.14	1.37	3.25
	Desirable	2.28	2.74	6.50
	Minimal	3.42	4.11	9.76
Serum Phosphate	Optimal	1.17	2.26	4.20
	Desirable	2.36	4.51	8.41
	Minimal	3.54	6.78	12.61

## Discussion

This research paper provides the findings of an investigation into the BV of 10 biochemistry analytes measured in human blood samples. The current research evaluated the analytical variance (CV_A_), index of individuality (II), and RCV for these components in a healthy adult population in Bangladesh. There are very few studies in the literature about the BV parameters of blood sugar, creatinine, urea, uric acid, chloride, calcium, potassium, magnesium, and inorganic phosphate.

In the present study, the CV_W_ for blood sugar, creatinine, sodium, chloride, and phosphate were calculated as 5.10 %, 4.64%, 0.69%, 0.75%, and 4.72% respectively. Falkeno et al. and Baral et al. reported higher CV_W_ for these parameters in the cat serum samples than our results [13]. The CVGs for blood sugar, urea, uric acid, sodium, potassium, chloride, calcium, magnesium, and phosphate were 10.70%, 31.47%, 23.52%, 1.95%, 9.74%, 2.56%, 4.64%, 9.96%, and 17.45%, respectively. Falkeno et al. and Baral et al. reported the CV_G_, which was lower in the cat serum sample than our finding [13,14].

The index of individuality (II) shows how well reference ranges are used to figure out what test results mean. If an individual's II is very low (0.6), then that person's test results should be evaluated in light of his or her prior test results rather than against arbitrary reference values. In the present study, nine serum biochemistry analytes (blood sugar, creatinine, urea, uric acid, sodium, potassium, chloride, magnesium, and phosphate) had low individuality, indicating that subject-based reference intervals are appropriate. Only one analyte (calcium) had high individuality, and, therefore, population-based reference intervals are more appropriate. Similar results found by Falkeno et al. reported that blood sugar, potassium, magnesium, and phosphate had low individuality in a cat blood sample [13]. In contrast to our result, Baral et al. reported that creatinine showed high index individuality in cat blood samples [14].

The RCV is a crucial metric in BV as well. RCV might be determined for numerous analytes by using BV data from a healthy population. RCV is the minimum difference between two consecutive patient analyses that must be met or surpassed in order to be considered statistically significant. Using RCV, findings from many measurements taken from the same patient that are all within the reference range but show a significant change are utilized to suggest a clinically significant shift in the patient's state. Blood sugar, calcium, magnesium, and phosphate RCVs were lower while creatinine and potassium RCVs were greater in cat serum samples compared to our results, as reported by Falkeno et al [13]. Setting quality standards for how well an analysis works is another thing that can be learned from data about biological variation. Using the atomic absorption spectroscopy method, estimates (minimum, desired, and ideal analytical objectives for imprecision, bias, and total error) were made for CV_W_, CV_G_, and CV_A_ (Table 4).

There are some limitations of our study. All analyses were done with the same apparatus and with reagents produced by the same company, which is another limitation. Moreover, the precision and accuracy of the findings may be affected by analytical variables (such as the experience of the instrument operator, the state of the environment, and the age and condition of the instruments used). Another limitation to determining the BV of the whole Bangladeshi population, more extensive studies at the national level with large sample sizes are needed.

## Conclusions

The goals of the study were to determine the biological variance of these clinical parameters and select the most appropriate test for a given clinical scenario. Nine serum biochemistry analytes (blood sugar, creatinine, urea, uric acid, sodium, potassium, chloride, magnesium, and phosphate) had low individuality, indicating that subject-based reference intervals are appropriate, only one analyte (calcium) had high individuality and, therefore, population-based reference intervals are more appropriate
